# Personal dispositions explain differences in physical health benefits of nature exposure: the role of restorativeness and affect

**DOI:** 10.3389/fpsyg.2024.1365512

**Published:** 2024-03-11

**Authors:** Claudia Russo, Luciano Romano, Giuseppina Spano, Annalisa Theodorou, Giuseppe Carrus, Stefano Mastandrea, Cinzia Angelini, Giovanni Sanesi, Angelo Panno

**Affiliations:** ^1^Experimental and Applied Psychology Laboratory, Department of Human Sciences, European University of Rome, Rome, Italy; ^2^Department of Education, Psychology, Communication Sciences, University of Bari Aldo Moro, Bari, Italy; ^3^Department of Education, Roma Tre University, Rome, Italy; ^4^Department of Soil, Plant and Food Sciences, University of Bari Aldo Moro, Bari, Italy

**Keywords:** nature connectedness, love and care for nature, place identity, positive and negative affect, physical wellbeing

## Abstract

**Introduction:**

Urbanization processes are constantly increasing, and most of the European population currently live in urban areas. Nevertheless, evidence is consistent in highlighting the positive association between nature exposure and human wellbeing, although individual differences might affect this association.

**Methods:**

The present study aimed to investigate the association among nature connectedness, conceptualized as Love and Care for Nature, place identity, and physical wellbeing, via restorativeness and positive and negative affect. A total of 312 visitors of an urban green area (i.e., Milan’s Parco Nord) participated in the study. They completed an anonymous questionnaire.

**Results:**

Findings showed that nature connectedness and place identity positively affect physical wellbeing, via restorativeness and positive affect, but not through the negative ones.

**Discussion:**

Results highlight the importance of the joint role of exposure to nature and individual differences in promoting wellbeing. This study offers implications for interventions aimed at enhancing individuals’ health through exposure to nature. Limitations of the study and future research developments are discussed.

## 1 Introduction

Urbanization levels are increasing dramatically throughout Europe, with 75% of the population currently living in urban areas (The World Bank, 2020). Considering Italy, the rate of people living in urban areas is around 72%, with a higher rate in Northern Italy, where the present study was carried out.

Urbanization processes significantly shape population health, producing mixed outcomes (Miao and Xiaogang, 2016). On the one hand, urban areas have several functions in our society because they represent the core in which technological and economic growth happens. Thus, urban residents can benefit from several public services and infrastructures (Abdul et al., 2020). Conversely, they might face other issues; for example, people might have difficulties accessing natural areas easily (Miao and Xiaogang, 2016). Within cities, homes and working spaces have become increasingly separated from green areas (D’Alessandro et al., 2015). However, nature has an important role in shaping people’s health (Wood et al., 2017). According to the “biophilia hypothesis,” humans have evolved with nature and, therefore, have an innate affinity with it. The need to establish a connection with nature represents an intrinsic facet of our biological heritage (Kellert and Wilson, 1995). Indeed, exposure to nature leads people to achieve states of restorativeness, i.e., the perception of “being away,” fascinated and totally absorbed by nature (Berto, 2014; Han, 2018; Spano et al., 2023). As a matter of fact, evidence supports the idea that people prefer natural environments (e.g., natural landscapes) over man-made environments (e.g., urban landscapes) (Berg et al., 2003; Capaldi et al., 2014; Dopko et al., 2014). Based on the “biophilia hypothesis,” two theories shed light on the association between contact with nature and people’s wellbeing, namely the Attention Restoration Theory (ART) (Kaplan and Kaplan, 1989) and the Stress Reduction Theory (SRT) (Ulrich et al., 1991). According to the ART, exposure to nature enables people to overcome mental fatigue and restore cognitive resources, such as directed attention. SRT instead states that contact with nature positively influences people’s affect by activating the parasympathetic nervous system, reducing stress levels (Jimenez et al., 2021). Thus, urban green areas, such as gardens and parks, represent an effective resource for both ecological and human health. Indeed, urban green spaces provide biodiversity, mitigate air pollution, and represent a source for people who live in urban areas to alleviate their stress levels and satisfy their inherent need to be connected with nature (Kabisch et al., 2017; Panno et al., 2020). Consistently, several previous studies empirically highlighted the positive impact on people’s health resulting from direct contact with urban natural areas (van den Bosch and Ode Sang, 2017). For instance, Carrus et al. (2017) showed that botanical gardens enhance the perceived restorativeness in their visitors, directly and indirectly via the perceived physical and psychological benefits of the visit. However, the authors noticed that this relationship varies across some respondents’ characteristics (e.g., family status).

Thus, individual differences, such as socio-demographic variables, but also dispositional and personality characteristics, might affect the association between natural areas and the benefits associated with nature’s exposure (Berto et al., 2018; Menardo et al., 2021). Among individual characteristics, a significant role might be played by connectedness to nature. Connectedness to nature can be conceptualized as a “an individual’s affective, experiential connection to nature” (Mayer and Frantz, 2004; p., 504). It has been operationalized in several different ways (Martin and Czellar, 2016); for example, some authors refer to it under the label of Nature Relatedness (Nisbet et al., 2008), or Connectivity with Nature (Dutcher et al., 2007). This difference in construct operationalization is driven by the focus posited on the emotional connectedness or the cognitive processes (Martin et al., 2020). In the present study, we specifically refer to Love and Care for Nature (Perkins, 2010), which pertains to individuals’ personal and explicit emotional connection with the natural world.

Connectedness to nature (broadly conceptualized) is a fairly stable trait over time (Martin et al., 2020). People differ in their levels of connectedness to nature, and this individual difference affects the motivation, emotions, and sense of restorativeness potentially enhanced by contact with nature (Mayer et al., 2009; Tam, 2013; Capaldi et al., 2014). In this regard, evidence is consistent in highlighting that people who reported higher scores in connectedness to nature are more prone to also report benefits from exposure to nature (Nisbet and Zelenski, 2011; Tam, 2013; Berto et al., 2018). For instance, individuals with high levels of connectedness to nature are more likely to experience nature’s restorative effects (Kaplan, 1995; Richardson et al., 2016), positive affect (Mayer et al., 2009), and vitality (Capaldi et al., 2014). Moreover, Howell et al. (2011) showed positive and significant relationships between connectedness to nature and different facets of wellbeing (i.e., emotional, psychological, and social wellbeing). Similarly, Dean et al. (2018) pointed out that people with higher levels of connectedness to nature were less likely to report symptoms of depression or anxiety and to report higher levels of physical health, assessed by the Body Mass Index (BMI).

Another individual factor that can potentially foster the link between exposure to green urban areas and people’s health is the place identity. Indeed, psychological theories of identity stress the idea that the self-concept is not only shaped by the inclusion of other humans in the self but also by the inclusion of other living species or ecosystems (Bragg, 1996). Place identity can be, therefore, conceptualized as a mix of concepts, memories, and ideas related to specific physical settings (Proshansky, 1983), which become an integral part of the self (Hidalgo and Hernandez, 2001). Place identity is built through an iterative process linked to a sense of belonging to a specific place (Twigger-Ross and Uzzell, 1996), reflecting the socio-cultural relationship with this place (Qazimi, 2014). Personal identification with a physical place involves place-related knowledge and feelings allocated in the declarative and autobiographical memory (Conway, 2005). According to Kneiz (2014), place identity is shaped by cognitive and affective components. The first refers to mental temporality, coherence, and reflection processes (Klein et al., 2004). The second one, instead, is related to the processes of attachment, closeness, and belongingness (Knez, 2005). Accordingly, people can think, reason, and remember a specific place that assumes a significant value because they are emotionally attached to that place (Knez and Eliasson, 2017). Thus, the association between nature exposure and benefits from exposure to nature should be influenced by the identity significance that the specific green area assumed for the person (Dean et al., 2018). However, the association between place identity and the positive effects of nature have been sparsely addressed. A few research highlighted a significant and positive relationship between place identity, emotion regulation (Ruiz and Hernández, 2014), and restorative potentials of nature (Knez and Eliasson, 2017; Morton et al., 2017). Finally, Knez et al. (2018) showed that place identity positively and significantly predicted the levels of wellbeing experienced by a group of people who live near an urban green area.

### 1.1 The present study

Based on the reviewed findings, it is possible to notice that the few studies available in the literature mainly focused on the direct associations between connection to nature, place identity, and benefits from nature exposure. Thus, the main aim of the present study was to contribute to overcoming this research gap, shedding light on the role of love and care for nature and place identity as individual factors in improving physical health. Furthermore, we deepened the underlying mechanisms in the relationship mentioned above, specifically focusing on the potential mediating role of restorativeness and positive and negative affect. We investigated these relationships by involving a sample of visitors to Parco Nord (PNM) a metropolitan peri-urban park located in the northern suburbs of Milan, a metropolitan city in northern Italy.

Specifically, on the one hand, based on previous studies that highlight direct positive associations between connectedness to nature and restorativeness (Richardson et al., 2016), positive affect (Mayer et al., 2009), or various facets of wellbeing (Howell et al., 2011; Dean et al., 2018), and on the other hand, the few studies that showed a positive link between place identity and benefits from exposure to natural areas (Knez and Eliasson, 2017; Morton et al., 2017; Knez et al., 2018), we formulated the following hypotheses:

*H1:* Love and care for nature would increase restorativeness and positive affect which in turn would lead to greater physical health.

*H2:* Place identity would increase restorativeness and positive affect, leading to greater physical health.

*H3:* Love and care for nature would lead to greater physical health through the reduction of negative affect.

*H4:* Place identity would lead to greater physical health through a decrement in negative affect.

## 2 Materials and methods

### 2.1 Procedures

The present study involved park visitors recruited during their stay at PNM. We employed a convenience sampling technique to achieve the goal of at least 10 participants for each selected data collection point in the park (for more details see Study area section).

Inclusion criteria for study participation were: (a) age of at least 18 years; (b) absence of cognitive impairment and/or mental or neurodegenerative illnesses. Before starting the interview, the researcher ensured that these inclusion criteria were met. If participants declared that they were under the age of 18 or they received a diagnosis of cognitive impairment and/or mental or neurodegenerative illness, the researcher did not proceed with the interview.

Park visitors were approached and invited to participate in the research. Each participant was assured that the questionnaire was anonymous, and that the data would be processed in aggregate form. Furthermore, the possibility to withdraw their participation at any time was explained, providing a contact for questions and/or feedback. Participants were individually interviewed by a well-trained interviewer at the same location where they were initially approached. Data were gathered via an online anonymous questionnaire. The interviewer was tasked with administering the questionnaire and logging participants’ responses using a tablet. At the end of the questionnaire, the interviewer documented the GPS coordinates of the precise location of the administration. The entire procedure typically lasted around 30 min. Data collection occurred between the 23rd of June and the 28th of July 2021. Informed consent was obtained from all participants. The study was approved by the institutional ethics committee of the European University of Rome, Italy (protocol n. 06/2021). All methods were performed in accordance with the Declaration of Helsinki and its later amendments.

### 2.2 Participants

The final sample involved in the study was composed of 312 visitors to Milan’s Parco Nord. Age ranged from 18 to 91 (*M* = 52.89; SD = 20.91). As reported in Table 1, of the total sample 176 (56.4%) were males. Concerning marital status, most participants were married/civilly married. Finally, concerning working status, most participants were retirees.

**Table 1:** Sociodemographic characteristics of the study sample.

	Variables	N	%
Sex			
	Males	176	56.4
	Females	136	43.6
Marital status			
	Married	125	40.1
	Living with their partner	39	12.5
	In a relationship but not living together	33	10.6
	Single	57	18.3
	Divorced	32	10.3
	Widower	26	8.3
Work status			
	Employee	65	20.8
	Self-employed	34	10.9
	Student	44	14.1
	Retiree	122	39.1
	Other	47	15.1

### 2.3 Study area

**Figure 1:**
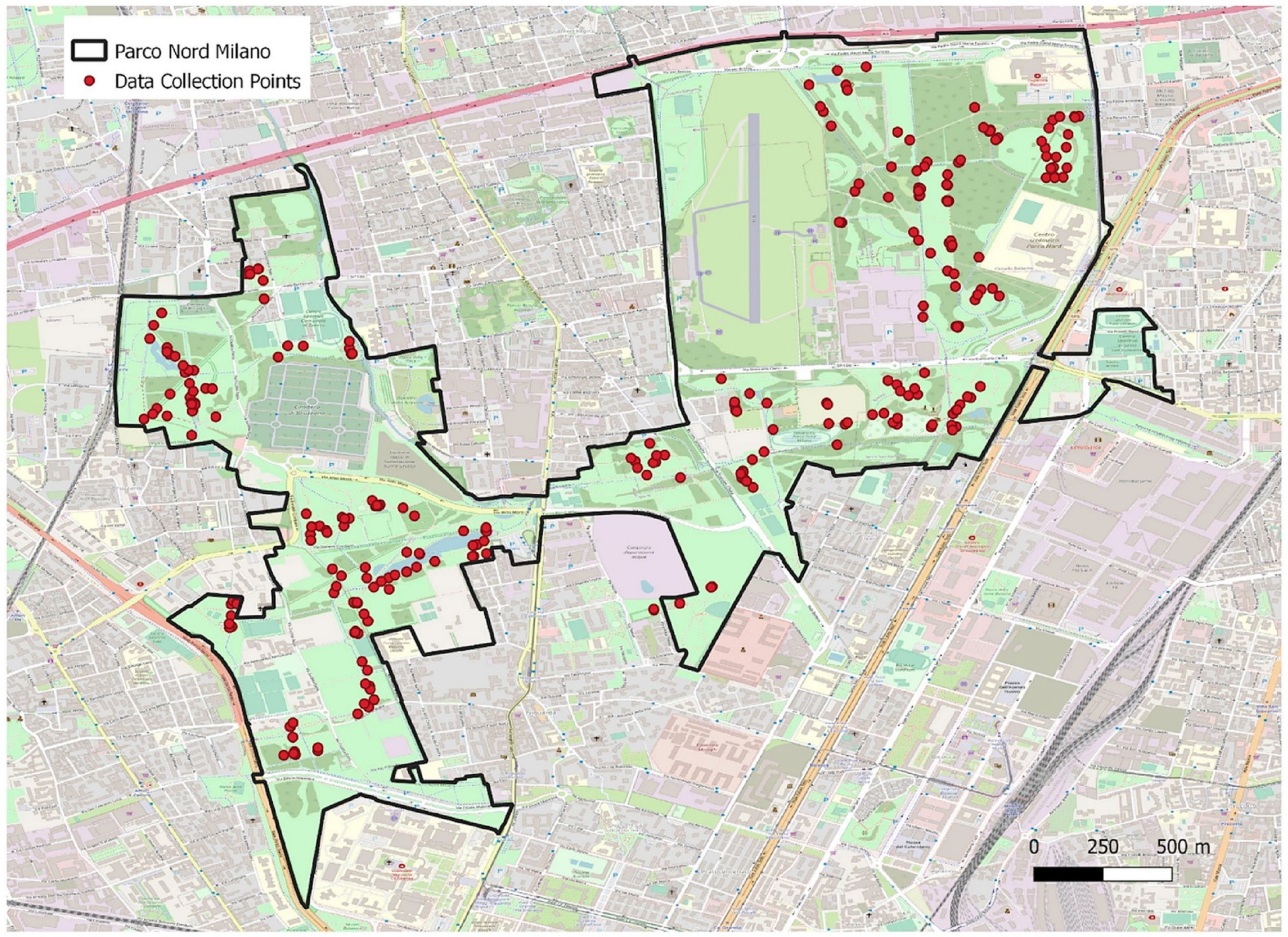
Map of data collection points in the study area (Parco Nord Milan, Italy). Reproduced from Spano et al. (2023), licensed under CC BY 4.0.

The study was conducted in a large urban green area situated in the metropolitan area of Milan within the Lombardy region of Northern Italy (coordinates: 45°53′71”N, 9°20′7″E). This area was chosen based on previous studies that highlighted the positive impact of this urban green area on wellbeing (Panno et al., 2017; Spano et al., 2023). The area covers 790 hectares in which there are forest plantations, tree rows, agricultural expanses, open spaces, infrastructure elements, and artificial installations (e.g., out-door gym). To properly represent the multiform characteristics of green spaces throughout the park, a total of 30 sampling points were randomly designated through the “Create Random Points” tool within the ArcMap software, using the study area as a spatial constraint (Figure 1). The park areas involved in the study were: (a) forest and agricultural areas, (b) meadows, (c) park with structures. At least 10 participants were recovered for each point.

### 2.4 Instruments

The questionnaire was composed of a set of sociodemographic questions and the following measures for a total of 44 items (in this final count, sociodemographic variables are included).

#### 2.4.1 Love and care for nature

A selection of five items from the Love and Care for Nature Scale (LCN) (Perkins, 2010) was used to measure love and care for nature. The items were selected among the ones with the highest factor loadings according to the original scale. Specifically, the items extracted from the original scale were: items 1, 2, 3, 4, and 6. The LCN is measured through a 7-points Likert scale (1 = Strongly disagree, 7 = Strongly agree). An item example is “I often feel a sense of awe and wonder when I am in un-spoilt nature.” In the current study, Cronbach’s alpha was 0.88 and McDonald’s omega was 0.89.

#### 2.4.2 Place identity

Two *ad hoc* items on a 5-points Likert scale (1 = Strongly disagree, 5 = Strongly agree) were created to evaluate place identity. The items are “This park means a lot to me” and “I am very fond of this park.” Cronbach’s alpha was 0.85 and McDonald’s omega was 0.86.

#### 2.4.3 Positive and negative affect

Positive and negative affect were assessed using the Italian validated version of the Positive and Negative Affect Schedule (PANAS) (Terraciano et al., 2003). In detail, PANAS encompasses 20 items and two sub-scales, one for positive affect and one for negative ones, both consisting of 10 items each and rated on a 5-points Likert scale (1 = At all, 5 = Very much). Participants were asked to answer a series of adjectives describing their emotions by thinking about how they feel when they are in the park. An example of positive affect is “Interested,” while an example of negative affect is “Anguished.” Cronbach’s alpha was 0.76 for positive affect and 0.81 for negative affect. McDonald’s omega was 0.78 for positive affect and 0.88 for negative affect.

#### 2.4.4 Restorativeness

To assess the restorative quality of the park, the Italian validated version of the Perceived Restorativeness Scale (PRS-11) (Pasini et al., 2014) was used. The scale comprises 11 items on a 5-points Likert scale (1 = At all, 5 = Very much). An item example is “This place is fascinating.” Cronbach’s alpha was 0.81 and McDonald’s omega was 0.83.

#### 2.4.5 Physical health

Two *ad hoc* items on a 5-points Likert scale (1 = Never, 5 = Always) were created to evaluate perceived physical health related to the park. The items are “When you visit the park, do you feel full of energy?” and “When you visit the park, do you find it easier to engage in physically demanding activities?”. Cronbach’s alpha was 0.71 and McDonald’s omega was 0.73.

### 2.5 Analysis plan

Preliminary descriptive statistics, such as mean, standard deviation, minimum, maximum, skewness and kurtosis, were performed using SPSS v.21.0 (Statistical Product and Service Solutions, Chicago, IL, USA) to verify the adequate normality of the used variables. As skewness and kurtosis of the PANAS negative affect scale were > 2, a non-normal distribution was assumed and Spearman correlations were performed to test the associations among the study variables.

To test the hypothesized mediation model, a Structural Equation Model (SEM) using Maximum Likelihood approach (ML) and with 5,000 resamples of bootstrapped estimates with a 95% confidence interval (CI) was performed by Mplus v. 8.3 (Muthén and Muthén, Los Angeles, CA, USA). The bootstrapping procedure provides non-symmetrical confidence intervals relevant to parameter estimates with non-normal sampling distributions, such as for variances and indirect effects. Besides, recent findings suggest that, compared to robust standard errors and the robust likelihood-based methods, bootstrap resampling still represents the best approach to use if the normality assumption is violated (Falk, 2018). Concerning the model specification, place identity and love and care for nature were the predictors, PANAS positive and negative affects, restorativeness were the mediators, and perceived physical health was the outcome.

Place identity and physical health were used as latent variables with their items (two for each construct) as observed variables. Furthermore, to avoid model non-identification and for parsimony purposes, the item parceling procedure (item aggregations) was modeled for love and care for nature, PANAS positive and negative affect, and restorativeness. Specifically, two parcels for each construct were used. The item parcels were randomly selected, and parcels had comparable reliabilities (Byrne, 1998). In detail, Cronbach’s alpha of the parcels was: 0.82 (items 6, 4, and 1 of the original scale; Perkins, 2010) and 0.79 (items 3 and 2) for love and care for nature; 0.63 (items 3, 5, 10, 6, and 8 of the original positive affect dimension, see Terraciano et al., 2003) and 0.61 for PANAS positive affect (items 9, 7, 1, 4, and 2 of the original positive affect dimension), 0.76 (items 1, 2, 7, 8, and 10 of the original negative affect dimension) and 0.73 (items 3, 4, 5, 6, and 9 of the original negative affect dimension) for PANAS negative affect; and 0.71 (items 2, 4, 5, 6, and 7 of the original scale; see Pasini et al., 2014) and 0.76 (items 1, 3, 8, 9, 10, and 11) for restorativeness. Respectively, McDonald’s omega of the parcels was: 0.84 and 0.80 for love and care for nature; 0.68 and 0.64 for PANAS positive affect 0.82 and 0.77 for PANAS negative affect; 0.73 and 0.77 for restorativeness.

**Figure 2:**
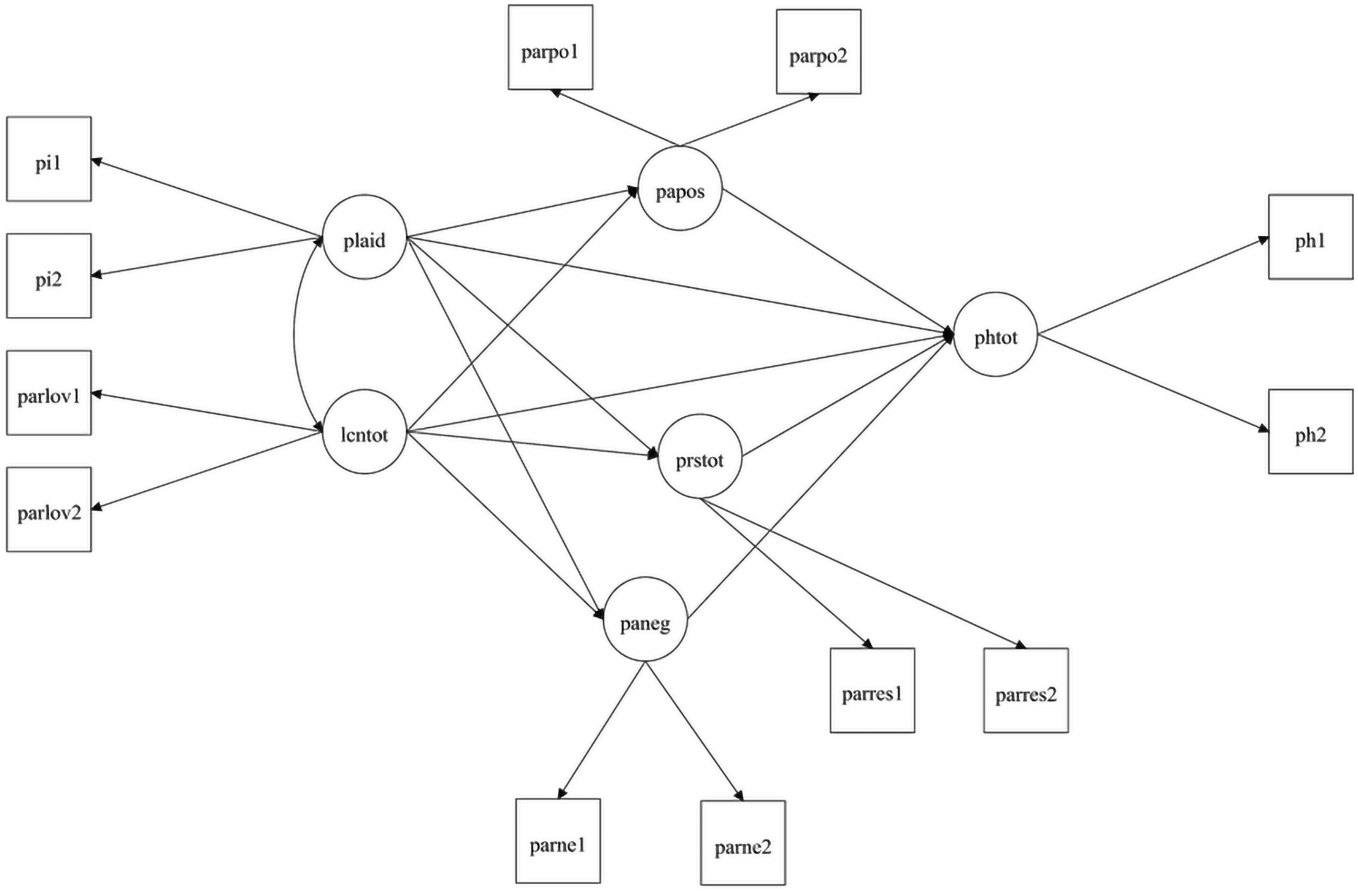
Hypothesized mediation model. Lcn, Love and care for nature; plaid, Place identity; papos, PANAS positive; paneg, PANAS negative; prstot, Restorativeness; phtot, Perceived physical health. Arrow from papos to paneg, arrow from restorativeness to papos, and arrow from restorativeness to paneg were not included in the figure for clarity purpose.

The following fit indexes were adopted to verify the goodness of fit of the hypothesized model: Chi-square test of exact fit (χ2), comparative fit index (CFI; >0.95 for good; >0.90 for acceptable), Tucker–Lewis index (TLI; >0.95 for good, >0.90 for acceptable), standardized root mean square residual (SRMR; <0.05 for good, <0.10 for acceptable), the root mean square error of approximation (RMSEA; <0.06 for good, <0.08 for acceptable) (Bentler and Bonnet, 1980; Hu and Bentler, 1999; McDonald and Ho, 2002). The hypothesized model is shown in Figure 2.

## 3 Results

### 3.1 Descriptive statistics and correlations

Table 2 shows means, standard deviations, minimum, maximum, skewness and kurtosis, and the correlation matrix depicting Spearman’s correlations among study variables.

**Table 2:** Descriptive statistics and Spearman correlation matrix.

	M	SD	Min	Max	SK	KU	2	3	4	5	6
1. LCN	30.55	4.27	15	35	−1.06	0.98	0.347^**^	0.399^**^	0.381^**^	−0.207^**^	0.309^**^
2. PI	8.27	1.65	4	10	−0.61	−0.48	-	0.440^**^	0.406^**^	−0.020	0.312^**^
3. REST	41.35	6.03	25	55	−0.34	0.02		-	0.473^**^	−0.046	0.441^**^
4. PANAS_P	32.88	6.37	12	47	−0.299	−0.01			-	−0.070	0.506^**^
5. PANAS_N	10.98	2.66	10	41	6.45	58.95				-	−0.109
6. PH	8.27	1.65	4	10	−0.00	−0.74					-

Raw data correlation matrix; **p < 0.01, LCN, Love and care for nature; PI, Place identity; REST, Restorativeness; PANAS_P, Panas positive affects; PANAS_N, Panas negative affects; PH, Perceived Physical Health.

Results highlight that love and care for nature is positively related to PANAS positive affect, place identity, restorativeness, and perceived physical health (*p* < 0.01). Besides, place identity is positively and significantly associated with restorativeness, PANAS positive affect, and perceived physical health (*p* < 0.01). Furthermore, restorativeness is positively and significantly associated with PANAS positive affect (*p* < 0.01), and both restorativeness and PANAS positive affect were positively and significantly related to perceived physical health (*p* < 0.01). Except for love and care for nature, which is negatively and significantly associated with PANAS negative affect (*p* < 0.01), no other significant relations emerged between PANAS negative affect and the other variables.

### 3.2 Mediation analysis results

**Figure 3:**
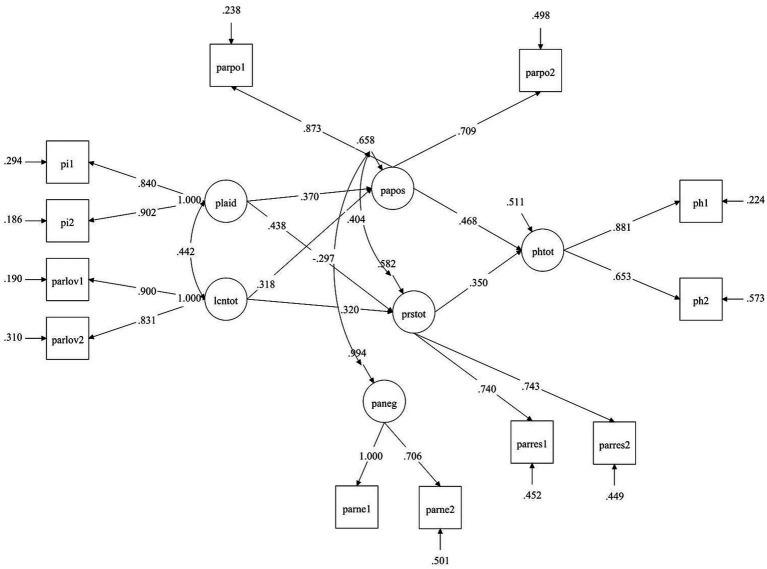
Mediation model results. Lcn, Love and care for nature; plaid, Place identity; papos, PANAS positive; paneg, PANAS negative; prstot, Restorativeness; phtot, Perceived physical health. For clarity purpose, only significant paths were reported in the figure.

The structural equation model (SEM) performed to examine the mediation of positive and negative affect and restorativeness in the relation among place identity, love and care for nature (predictors), and perceived physical health (outcome), shows a good fit to the data. In detail, χ2 (40) = 98.422, *p* < 0.01; root mean square error of approximation (RMSEA) = 0.068; standardized root mean square residual (SRMR) = 0.033; comparative fit index (CFI) = 0.963; Tucker–Lewis index (TLI) = 0.939. Concerning the mediations among the study variables (see Figure 3), results show that the relation between love and care for nature and perceived physical health (*β* = 0.015, *p* > 0.05, 95% CI: [−0.121, 0.146]) goes through PANAS positive affect (*β* = 0.149, *p* < 0.05, 95% CI: [0.062, 0.254]) and restorativeness (*β* = 0.112, *p* < 0.05, 95% CI: [0.042, 0.209]), but not through PANAS negative affect (*β* = −0.007, *p* > 0.05, 95% CI: [−0.028, 0.009]). Similarly, place identity is related to perceived physical health (*β* = −0.060, *p* > 0.05, 95% CI: [−0.214, 0.080]) through PANAS positive affect (*β* = 0.173, *p* < 0.01, 95% CI: [0.079, 0.286]) and restorativeness (*β* = 0.153, *p* < 0.05, 95% CI: [0.058, 0.280]), but not via PANAS negative affect (*β* = −0.004, *p* > 0.05, 95% CI: [−0.033, 0.031]).

Table 3 summarizes the statistical significance of standardized effects (total, indirect paths, total indirect, and direct) and their relative bootstrap 95% CI.

**Table 3:** Results of mediation with standardized effects and bootstrap 95% CI.

Effects	Physical health [95% CI]
Love and care for nature	
Total	0.269*** [0.131, 0.404]
Indirect via PANAS positive	0.149* [0.062, 0.254]
Indirect via PANAS negative	−0.007 [−0.028, 0.009]
Indirect via Restorativeness	0.112* [0.042, 0.209]
Total indirect	0.253*** [0.152, 0.367]
Direct	0.015 [−0.121, 0.146]
Place identity	
Total	0.262*** [0.138, 0.386]
Indirect via PANAS positive	0.173** [0.079, 0.286]
Indirect via PANAS negative	−0.004 [−0.003, 0.031]
Indirect via Restorativeness	0.153* [0.058, 0.280]
Total indirect	0.323*** [0.214, 0.457]
Direct	−0.060 [−0.214, 0.080]

* *p* ≤ 0.05; ** *p* ≤ 0.01, *** *p* ≤ 0.001.

## 4 Discussion

The present study aimed to provide insights into the indirect effects of connectedness to nature, conceptualized as love and care for nature, and place identity on physical wellbeing, through restorativeness and positive and negative affect. In detail, we hypothesized that nature connectedness and place identity enhance restorative states and positive affect, and this in turn would lead to higher levels of physical wellbeing. Moreover, we also hypothesized that nature connectedness and place identity would lead to greater levels of physical wellbeing, via the reduction of negative affect.

Our findings partially confirmed our initial hypotheses. Consistently with the “biophilia hypothesis,” connectedness to nature promotes physical wellbeing, through restorativeness and positive emotions (H1). People who are highly affiliated with nature are more likely to feel an affective, other than cognitive, bond with the surrounding nature (Mayer and Frantz, 2004). Thus, connectedness to nature represents a catalyst in the promotion of positive emotions and states of restorativeness during the interaction with natural environments, enhancing physical wellbeing. Our results are in line with several previous studies that highlighted the role of connectedness to nature in promoting human wellbeing (Mayer and Frantz, 2004; Hinds and Sparks, 2008; Cervinka et al., 2011; Nisbet and Zelenski, 2011; Pensini et al., 2016), and extend them by shedding the light to the mechanisms behind this association. Moreover, adopting the psychodynamic understanding proposed by Cervinka et al. (2011), it is possible to assume that connectedness to nature represents a personal resource that enables individuals to take advantage of the benefits of staying in nature, coping with stressors and experiencing positive emotions, so much so that they can feel “physically alive.”

Additionally, our study highlighted that place identity is positively and significantly associated with wellbeing via the enhancement of positive affect and restorative effects (H2). The identification with a specific place entails place-related knowledge and feelings allocated in the declarative and autobiographical memory (Conway, 2005; Knez et al., 2018). It implies that people who include PNM in their self will again feel all the positive emotions allocated in their memory every time they have the chance to be there (Kihlstrom and Klein, 1994). Moreover, since they are cognitively and emotionally attached to PNM, their experience in the park will be characterized by high levels of the perception of “being away” and fascinated (Korpela and Hartig, 1996). This process will lead, in turn, to a sense of wellness.

Contrary to our expectations, neither love and care for nature nor place identity are related to wellbeing via the reduction of negative affect (H3, H4). In explaining these findings, it is noteworthy to consider that previous findings on the link between nature connectedness and negative emotions are inconsistent. Indeed, on the one hand, previous research highlighted that connectedness with nature is significantly related to negative affect (Dean et al., 2018) or non-adaptive emotion regulation strategies (Richardson et al., 2016). On the other hand, several other studies only reported a significant link between connectedness to nature and positive emotions (Mayer et al., 2009; Nisbet and Zelenski, 2011; Zelenski and Nisbet, 2012) or adaptive emotion regulation strategies (Fido et al., 2020; Bakir-Demir et al., 2021). Furthermore, the literature on place identity is consistent in considering that place attachment, which represents the affective dimension of place identity, develops for those places in which the person feels comfortable and secure (Hidalgo and Hernandez, 2001). Thus, it is plausible to assume that place identity enhances all the (positive) feelings and emotions associated with staying in a perceived safe place (Zheng et al., 2019). Although based on both our and previous findings it might seem that connectedness to nature and place identity might not be enough as a sole filter for mitigating negative outcomes (Bakir-Demir et al., 2021), we might instead adopt a positive framework. It is possible to speculate that both these individual factors bolster human wellbeing by relying on a promotion system strictly linked with personal growth dimensions.

In interpreting these findings, six main limitations must be acknowledged. First, the cross-sectional nature of the present study did not allow us to capture the causality of the relationships among the variables. Second, the relatively small convenience sample prevents the possibility of generalizing the findings. However, in order to partially face this limit, we used the bootstrap method to compute the confidence intervals (Schoemann et al., 2017). Third, we did not obtain information regarding the amount of time people spent in the park. Fourth, for the sake of questionnaire brevity, we used a selection of items to measure some constructs (i.e., love and care for nature, place identity, and physical wellbeing). The use of full-version scales and the adoption of a multimethod approach (e.g., measuring physical wellbeing with body indexes) could give greater solidity to the results obtained. Fifth, the standardized beta values obtained in the mediation model are relatively weak, considering that the closer the values are to one, the stronger the relationship is Pallant (2020). Finally, the internal consistency of PANAS positive affect parcels is low 0.70. However, it is noteworthy to point out that Cronbach’s alpha higher than 0.60 is usually considered acceptable (Taber, 2018).

## 5 Conclusion

Despite these limits, to the best of our knowledge, this is the first study that addressed the role of nature connectedness and place identity in promoting physical wellbeing, shedding light on the underlying indirect effects through restorativeness and affects.

Considering the current global challenges, such as the growing urbanization and the negative effects of climate change, scholars are increasingly interested in deepening the crucial role of urban green areas in mitigating the harmful effects of these threats on human health and wellbeing. Evidence in this direction is even more needed if focusing on the impact of urban green spaces in large metropolitan cities like Milan. Moreover, our study paves the way for the importance of pointing out the role of individual differences in mitigating or enhancing the effects of exposure to nature. Indeed, the joint investigation of both the role of exposure to nature and individual differences in promoting wellbeing makes it possible to define interventions that make people more sensitive to the beneficial effects that nature can offer them.

## Data availability statement

The raw data supporting the conclusions of this article will be made available by the authors, without undue reservation.

## Ethics statement

The study was approved by the institutional ethics committee of the European University of Rome, Italy (protocol n. 06/2021). The studies were conducted in accordance with the local legislation and institutional requirements. The participants provided their written informed consent to participate in this study.

## Author contributions

CR: Writing – original draft, Writing – review & editing. LR: Formal analysis, Writing – original draft, Writing – review & editing. GSp: Conceptualization, Data curation, Investigation, Methodology, Writing – review & editing. AT: Data curation, Investigation, Methodology, Writing – review & editing. GC: Data curation, Funding acquisition, Investigation, Methodology, Project administration, Writing – review & editing. SM: Data curation, Funding acquisition, Investigation, Methodology, Project administration, Writing – review & editing. CA: Data curation, Funding acquisition, Investigation, Methodology, Project administration, Writing – review & editing. GSa: Conceptualization, Data curation, Funding acquisition, Investigation, Project administration, Supervision, Writing – review & editing. AP: Conceptualization, Data curation, Funding acquisition, Investigation, Methodology, Project administration, Supervision, Writing – review & editing.
